# Antiviral Activity of Olanexidine-Containing Hand Rub against Human Noroviruses

**DOI:** 10.1128/mbio.02848-21

**Published:** 2022-03-17

**Authors:** Khalil Ettayebi, Wilhelm Salmen, Kaoru Imai, Akifumi Hagi, Frederick H. Neill, Robert L. Atmar, B. V. Venkataram Prasad, Mary K. Estes

**Affiliations:** a Department of Molecular Virology and Microbiology, Baylor College of Medicine (BCM), Houston, Texas, USA; b Naruto Research Institute, Research and Development Center, Otsuka Pharmaceutical Factory, Inc., Naruto, Tokushima, Japan; c Department of Frontier Science for Advanced Environment, Graduate School of Environmental Studies, Tohoku University, Miyagi, Japan; d Department of Medicine, BCM, Houston, Texas, USA; e The Verna and Marrs McLean Department of Biochemistry and Molecular Biology, Baylor College of Medicine, Houston, Texas, USA; McMaster University

**Keywords:** HuNoV, antiviral, virucidal activity, olanexidine, OLG-HR, HIEs, organoids, IntestiCult media, noroviruses

## Abstract

Human norovirus (HuNoV) is the leading cause of epidemic and sporadic acute gastroenteritis worldwide. HuNoV transmission occurs predominantly by direct person-to-person contact, and its health burden is associated with poor hand hygiene and a lack of effective antiseptics and disinfectants. Specific therapies and methods to prevent and control HuNoV spread previously were difficult to evaluate because of the lack of a cell culture system to propagate infectious virus. This barrier has been overcome with the successful cultivation of HuNoV in nontransformed human intestinal enteroids (HIEs). Here, we report using the HIE cultivation system to evaluate the virucidal efficacy of an olanexidine gluconate-based hand rub (OLG-HR) and 70% ethanol (EtOH_70%_) against HuNoVs. OLG-HR exhibited fast-acting virucidal activity against a spectrum of HuNoVs including GII.4 Sydney[P31], GII.4 Den Haag[P4], GII.4 New Orleans[P4], GII.3[P21], GII.17[P13], and GI.1[P1] strains. Exposure of HuNoV to OLG-HR for 30 to 60 s resulted in complete loss of the ability of virus to bind to the cells and reduced *in vitro* binding to glycans in porcine gastric mucin. By contrast, the virucidal efficiency of EtOH_70%_ on virus infectivity was strain specific. Dynamic light scattering (DLS) and electron microscopy of virus-like particles (VLPs) show that OLG-HR treatment causes partial disassembly and possibly conformational changes in VP1, interfering with histo-blood group antigen (HBGA) binding and infectivity, whereas EtOH_70%_ treatment causes particle disassembly and clumping of the disassembled products, leading to loss of infectivity while retaining HBGA binding. The highly effective inactivation of HuNoV infectivity by OLG-HR suggests that this compound could reduce HuNoV transmission.

## INTRODUCTION

Human noroviruses (HuNoVs) are a major cause of nonbacterial acute gastroenteritis in all age groups worldwide. They are classified, based on nucleotide diversity in the viral RNA-dependent RNA polymerase (RdRp) (P type) and the capsid (open reading frame 2 [ORF2]) sequences, into 10 genogroups, 49 genotypes, and 60 P types (1). According to the CDC, an estimated 685 million HuNoV cases and 212,000 deaths occur worldwide annually, with an economic impact of over $60 billion in health care and societal costs in the United States alone (2). Most HuNoV outbreaks occur in semiclosed environments, such as schools, hospitals, and social health centers (3, 4). The health care burden associated with HuNoV results in part from poor hand hygiene and a lack of antiseptics proven to be effective against HuNoVs. Even 50 years since HuNoV was discovered, there are still no effective therapies or licensed vaccines against norovirus.

A significant number of disinfectants and sanitizers are currently available to hinder viral spread, and several factors can influence their effectiveness against specific viruses. Enveloped viruses, including influenza and coronavirus, are more susceptible to inactivation by disinfectants (5–7). Lipophilic disinfectants can interfere with the viral envelope and significantly reduce virus infectivity. In contrast, small nonenveloped viruses are generally more resistant and their inactivation requires denaturation of viral capsid (8) or disruption of the viral genome (9). Therefore, it is generally more difficult to prevent replication of small nonenveloped viruses, such as HuNoV and poliovirus, and several commonly available disinfectants do not sufficiently inactivate infectivity (10–12).

Olanexidine gluconate (1.5% Olanedine; Otsuka Pharmaceutical Factory, Tokushima, Japan), a new antiseptic agent currently only commercially available in Japan, is demonstrated to have a large antimicrobial spectrum against Gram-positive and Gram-negative bacteria, but its virucidal activity against nonenveloped viruses using cultivation methods has not been evaluated previously (13–16). Olanedine antiseptic solution efficacy was evaluated in a randomized, controlled clinical trial in Japan, and surgical site infections following gastrointestinal surgery occurred less frequently with its use than with the use of commercial antiseptics povidone-iodine and chlorhexidine (15, 17, 18). Its bactericidal efficacy as a topical antiseptic led to its proposed use for preoperative skin preparation prior to surgery (15) and as an environmental disinfectant for the prevention of nosocomial transmission of enveloped viruses, including influenza A viruses, respiratory syncytial viruses, and coronaviruses (19). Here, we tested the effectiveness, and explored the mechanism of action of olanexidine gluconate-containing hand rub (OLG-HR) on HuNoV infectivity.

## RESULTS

### OLG-HR and EtOH_70%_ mixed with neutralizer were not cytotoxic to jejunal HIE monolayer cultures.

**FIG 1:**
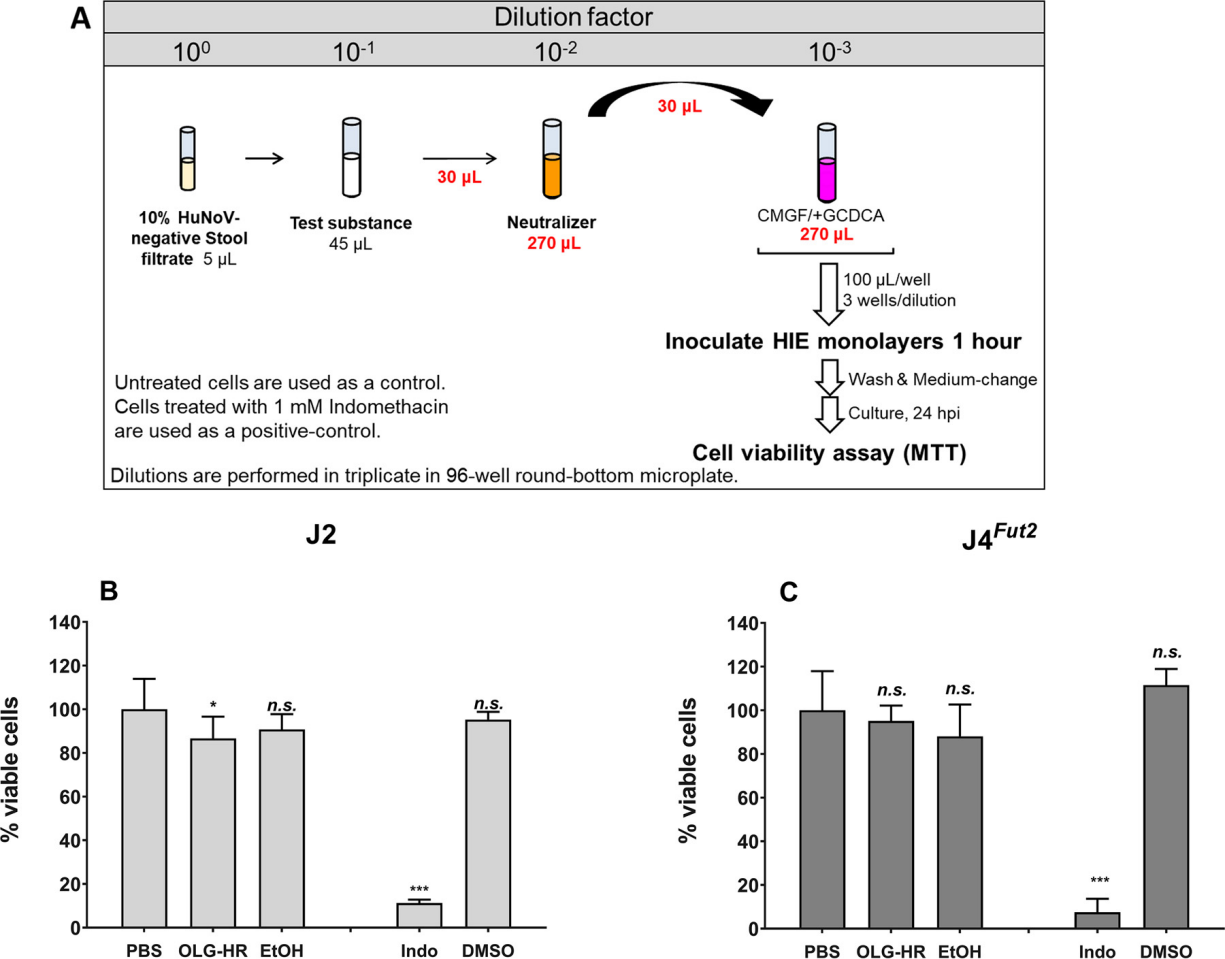
Virucidal agents, OLG-HR and EtOH, are not cytotoxic to jHIE monolayers. (A) Schematic experimental design. Cell viability of J2 (B) or J4*^Fut2^* (C) HIE monolayers, expressed as percentage relative to PBS-treated monolayers, was determined by MTT assay. PBS and indomethacin treatments were used as negative and positive controls, respectively. Each experiment was performed twice. Compiled data represent the mean of 6 wells for each treatment. Error bars denote standard deviation. Asterisks indicate significant difference in percentage of viable cells at the indicated treatment compared to those of the PBS control. Significance was determined using one-way ANOVA with Dunnett multicomparison test. *, *P* < 0.05; ***, *P* < 0.001; n.s., not significant.

We first investigated whether the test materials (OLG-HR or 70% ethanol [EtOH_70%_]) exhibit cytotoxicity following inactivation with neutralizer in the human intestinal enteroid cultures using the 3-[4,5-dimethylthiazole-2-yl]-2,5-diphenyltetrazolium bromide (MTT) assay. After a 24-h incubation, the cell monolayers, treated with either test material, did not show significant signs of cytotoxicity as observed under IX73 inverted microscope (see Fig. S1 in the supplemental material). Cell viability measured by the MTT assay showed 87% and 91% viability for OLG-HR- and EtOH_70%_-treated J2 human intestinal enteroid (HIE), respectively (Fig. 1A). OLG-HR showed slight cytotoxicity, while reduction in cell viability was not significant with EtOH_70%_ treatment. In contrast, OLG-HR showed no significant cytotoxicity on J4*^Fut2^* HIE cultures (95% viable cells), but only 88% viability was observed with EtOH_70%_ (Fig. 1B). Treatment with 1 mM indomethacin (positive control) showed a significant reduction in cell viability for both HIE cultures, while dimethyl sulfoxide (DMSO) vehicle-treated cultures did not show any cytotoxicity compared to that of the phosphate-buffered saline (PBS)-treated monolayers (Fig. 1).

10.1128/mBio.02848-21.1 FIG S1 Virucidal agents, OLG-HR and EtOH_70%_, did not significantly affect cell viability of HIEs. HIE cell monolayers, treated with either test material, were observed after 24 h incubation by bright field on Olympus IX73 inverted microscope. Scale bar, 20 μm. Download FIG S1, TIF file, 0.6 MB. Copyright © 2022 Ettayebi et al. 2022 Ettayebi et al. https://creativecommons.org/licenses/by/4.0/ This content is distributed under the terms of the Creative Commons Attribution 4.0 International license.

### Evaluation of neutralizer efficiency.

**FIG 2:**
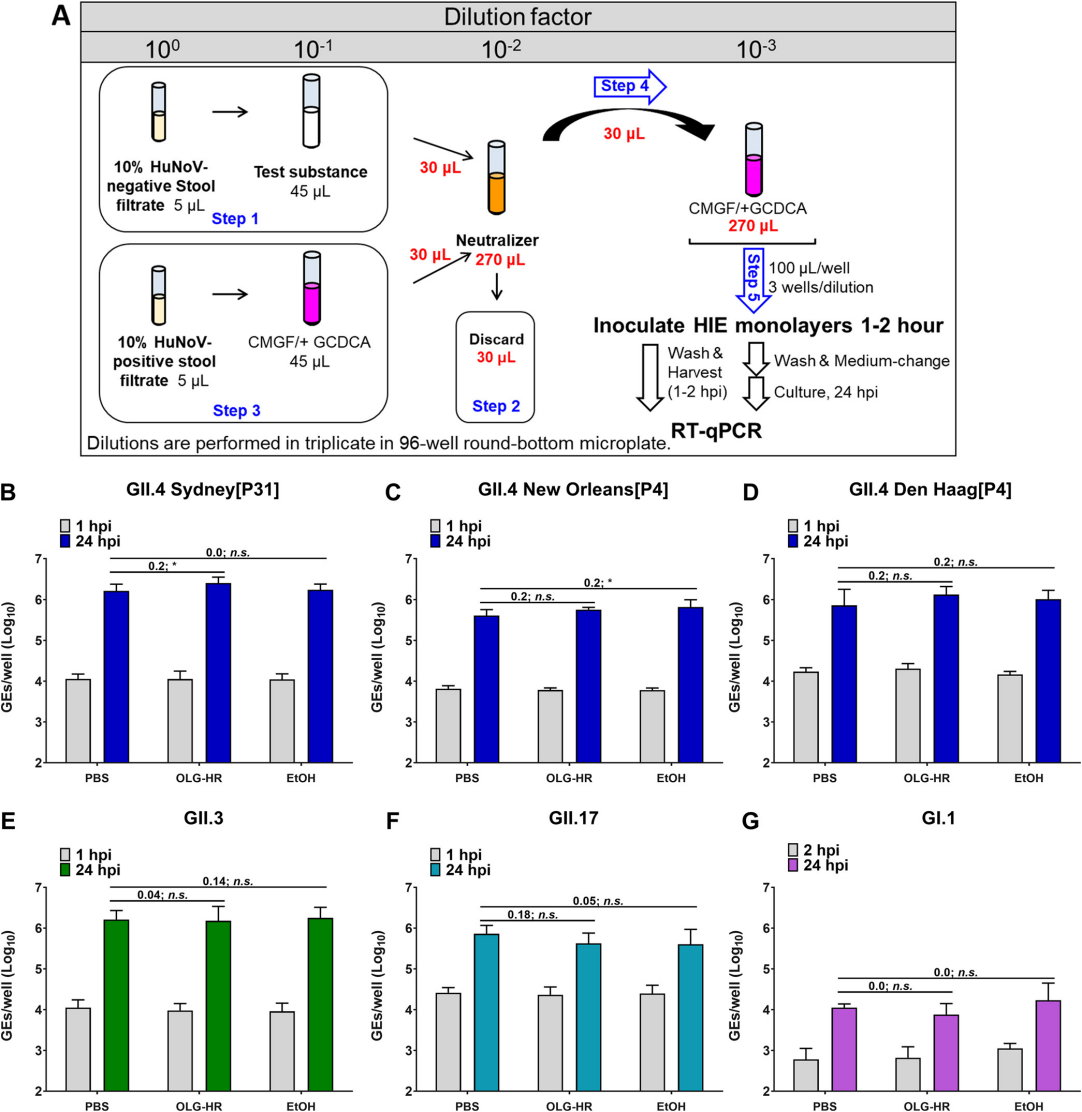
OLG-HR neutralizer does not affect HuNoV replication. Neutralizer efficiency was evaluated with GII.4 variants and GII.3 in J2 and with GII.17 and GI.1 in J4*^Fut2^* HIE culture monolayers. (A) Schematic experimental design. (B to G) Viral replication of HuNoV strains after each treatment. Compiled data from two experiments are presented. Error bars denote standard deviation (*n* = 12). Values above the bars represent log_10_ difference in net replication between conditions ([Δ24 hpi–1 hpi with treatment) − (Δ24 hpi–1 hpi with PBS)]. Significance was determined using one-way ANOVA followed by Dunnett’s test (*, *P* < 0.05; n.s., not significant).

The effect of the neutralizer to deactivate the virucidal agents was determined by exposing the OLG-HR or EtOH_70%_ test materials to the neutralizer prior to mixing with the HuNoV-positive stool filtrate. Viral infectivity was then assessed on J2 (for GII.4 variants and GII.3) or J4*^Fut4^* (for GII.17 and GI.1) HIE monolayers. A 0.5-log_10_ increase in genome equivalents (GEs) after 24 hours postinfection (hpi) relative to the amount of genomic RNA detected at 1 hpi (after removal of the virus inoculum and two washes of the monolayers to remove unbound virus) was set as a threshold to indicate successful viral replication (20). All HuNoV strains tested successfully infect jejunal HIE (jHIE) monolayers, and viral replication was successfully attained compared to the PBS control under each treatment condition (Fig. 2). These results indicate that the neutralizer effectively quenched the antiviral activity of the antiseptic agents and did not significantly adversely affect viral infectivity.

### OLG-HR and EtOH_70%_ have significant effects on HuNoV replication.

**FIG 3:**
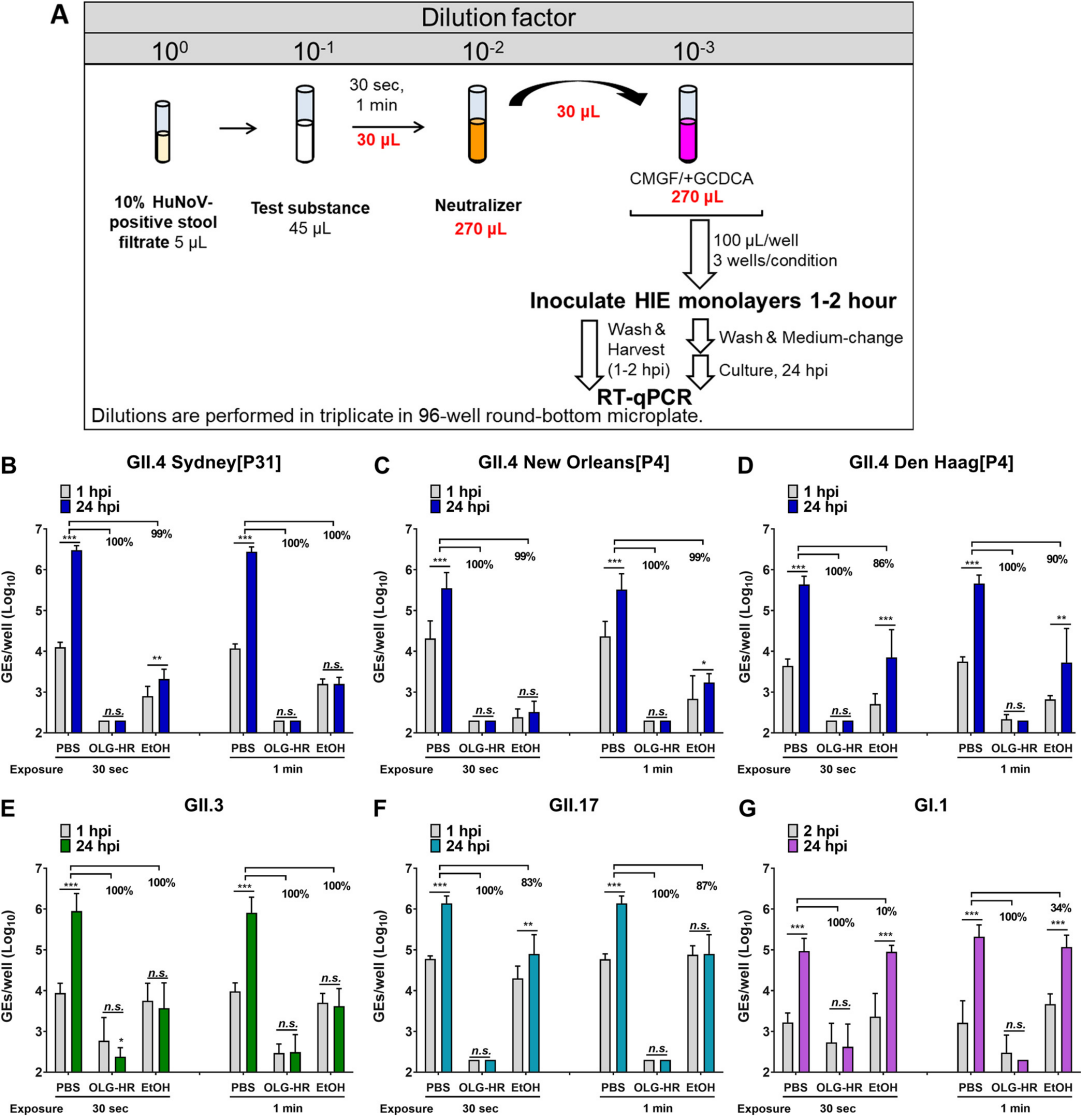
Virucidal agents, OLG-HR and EtOH, inhibit HuNoV replication. jHIE monolayers were inoculated with each virus previously exposed for 30 s or 1 min to the test substance and diluted with neutralizer. (A) Schematic experimental design. (B to G) Viral replication of HuNoV strains after each treatment. Compiled data of two experiments represent the geometric mean of 12 wells. Error bars denote standard deviation (*n* = 12). Values represent percentage of fold reduction in net viral replication in each condition relative to PBS control. Significance of net replication (Δ24 hpi–1 hpi) was determined using Dunnett’s multiple comparison test (*, *P* < 0.05; **, *P* < 0.01; ***, *P* < 0.001; n.s., not significant).

We next evaluated the effect of a 30-s or 1-min exposure to the OLG-HR, EtOH_70%_, or PBS (control) treatment of GII.4 Sydney[P31], GII.4 Den Haag[P4], GII.4 New Orleans[P4], GII.3[P21], GII.17[P13], or GI.1[P1] HuNoV stool filtrates. The effect of each virucidal solution was stopped by adding neutralizer after the indicated time point, and the viral infectivity was assessed on jHIE monolayers. OLG-HR treatment resulted in a complete loss of the ability of GII.17 or all GII.4 variants to bind to the cells. In fact, no viral signal was observed at 1 to 2 hpi or 24 hpi with both exposure times (30 s and 1 min) for GII.4 Sydney[P31], GII.4 Den Haag[P4], GII.4 New Orleans[P4], and GII.17[P13] (Fig. 3B to D and F); some virus binding was observed with GII.3 and GI.1 infections in both time point exposures, but each virus failed to replicate after 24 hpi (Fig. 3E and G).

In the EtOH_70%_ treatment groups, the virus retained the ability to bind to the jHIE cell monolayers with both time point exposures. While EtOH_70%_ treatment for 30-s and 1-min exposures resulted in a ≥99% loss of infectivity of GII.4 Sydney[P31], GII.4 New Orleans[P4], and GII.3, and a 1-min exposure led to loss of GII.17 infectivity, net viral replication was significantly reduced for GII.4 Den Haag[P4] (86% and 90% with 30-s and 1-min exposure, respectively), GII.17 (83% with 30-s exposure), and GI.1 (10% and 34% with 30-s and 1-min exposure, respectively). These results suggest that the virucidal activities of OLG-HR and EtOH_70%_ on HuNoV infectivity act through different mechanisms.

### OLG-HR inhibits glycan binding to HuNoV.

**FIG 4:**
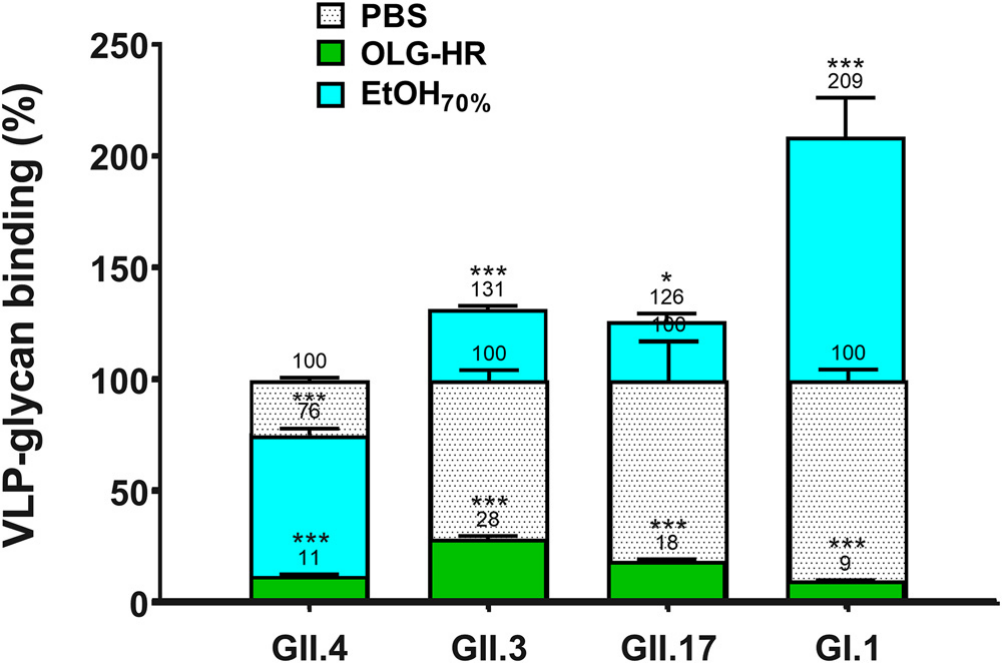
VLP-glycan binding assay. PGM binding to VLPs was determined by incubating OLG-HR-treated GII.4 Sydney, GII.3, GII.17, or GI.1 VLPs with PGM-coated plates, followed by VLP detection with guinea pig polyclonal antiserum to GII.4_Sydney 2012. Values on bars represent the glycan binding percentage relative to PBS control. Error bars denote standard deviation (*n* = 3). Asterisks indicate significant difference between treatment conditions and PBS (***, *P* < 0.001; *, *P *< 0.05).

The virucidal activity results indicate that OLG-HR and EtOH_70%_ affect the ability of some of the virus strains to bind to the cells. To investigate whether these virucidal agents interrupt the binding of the virus to histo-blood group antigens (HBGA), we performed virus-like particle (VLP)-glycan binding assays with OLG-HR-, EtOH_70%_-, and PBS-treated VLPs (Fig. 4; see also Table S2 in the supplemental material). Results indicate that 1-min exposure of GII.4, GII.3, GII.17, and GI.1 VLPs to OLG-HR led to a sharp reduction in binding to pig gastric mucin (PGM) glycans (Fig. 4; green bars), while EtOH_70%_ treatment showed a 2-fold increase (for GI.1) or significant alteration (for GII.4, GII.3, and GII.17) in glycan binding (Fig. 4; cyan bars).

10.1128/mBio.02848-21.4 TABLE S2 Absolute values (absorbance at 450 nm) of VLP-glycan binding. Download Table S2, DOCX file, 0.01 MB. Copyright © 2022 Ettayebi et al. 2022 Ettayebi et al. https://creativecommons.org/licenses/by/4.0/ This content is distributed under the terms of the Creative Commons Attribution 4.0 International license.

### OLG-HR and EtOH_70%_ induce aggregation of virus-like particles.

**FIG 5:**
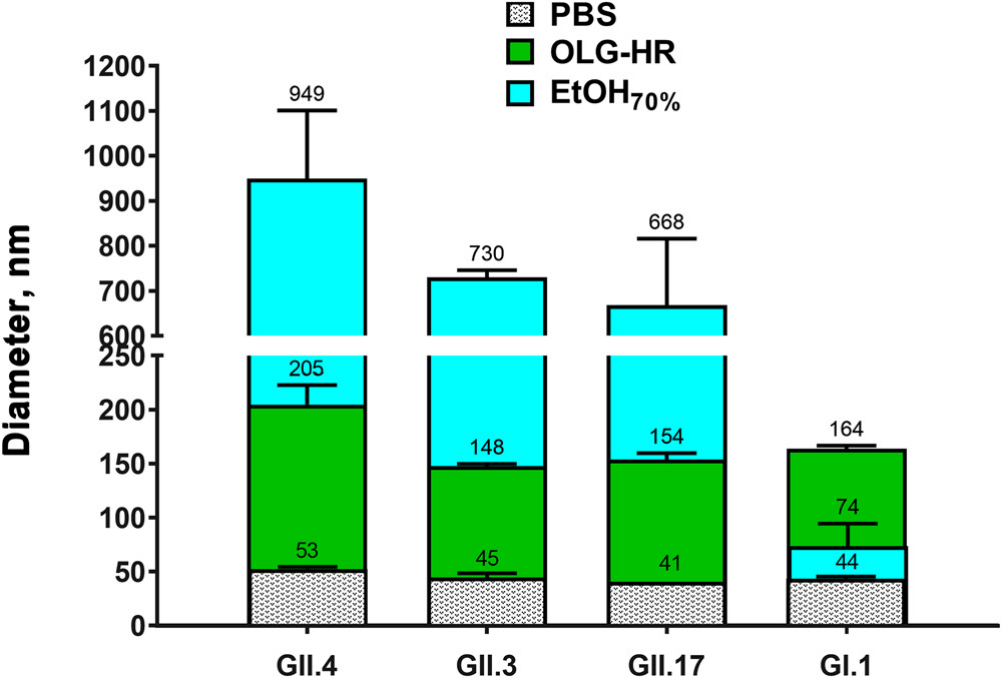
Dynamic light scattering of VLPs after OLG-HR or ethanol exposure. Average diameters of treated GII.4 Sydney, GII.3, GII.17, and GI.1 VLPs for each condition were calculated using Zetasizer software. VLP samples in PBS were incubated at a ratio of 1:9 with the indicated treatment for 1 min at room temperature and then diluted 1:10 in PBS to get a final VLP concentration of 200 nM in PBS. All experiments were performed in triplicates (*n* = 3) using standard settings (refractive index, 1.335; viscosity, 0.9; temperature, 25°C).

To understand the mechanism by which OLG-HR and EtOH_70%_ exert their virucidal activities on HuNoV, we analyzed treated HuNoV VLPs using dynamic light scattering (DLS) and electron microscopy. The hydrodynamic diameters of treated or untreated GII.4 Sydney[P31], GII.3, GII.17, and GI.1 VLPs were measured using DLS on a Zetasizer Nano instrument (Malvern Instruments, UK). PBS-control treated VLPs showed diameters of less than 50 nm (Fig. 5) with complete size distribution profiles presented in Fig. S2 in the supplemental material. After OLG-HR treatment, the hydrodynamic diameters moderately increased by 3- to 4-fold for GII.4, GII.3, GII.17, and GI.1 compared to diameters of PBS-treated VLPs. However, EtOH_70%_ treatment led to 16- to 17-fold increases in diameters for GII.4, GII.3, and GII.17, and a 1.4-fold increase in diameter for GI.1. These results indicate that while both OLG-HR and EtOH_70%_ treatments cause aggregation, the effects are larger with EtOH_70%_ treatment.

10.1128/mBio.02848-21.2 FIG S2 Dynamic light scattering size distributions of VLPs after OLG-HR or EtOH_70%_ exposure. Particle size distributions of treated GII.4 Sydney, GII.3, GII.17, and GI.1 VLPs for each condition were calculated using Zetasizer software, with the *y* axis indicating percent fraction of particle distribution based on volume. VLP samples in PBS were incubated at a ratio of 1:9 with the indicated treatment for 1 minute at room temperature and then diluted 1:10 in PBS to get a final VLP concentration of 200 nM in PBS. All experiments were performed in triplicate (*n* = 3) using standard settings (refractive index, 1.335; viscosity, 0.9; temperature, 25°C). Download FIG S2, TIF file, 0.2 MB. Copyright © 2022 Ettayebi et al. 2022 Ettayebi et al. https://creativecommons.org/licenses/by/4.0/ This content is distributed under the terms of the Creative Commons Attribution 4.0 International license.

**FIG 6:**
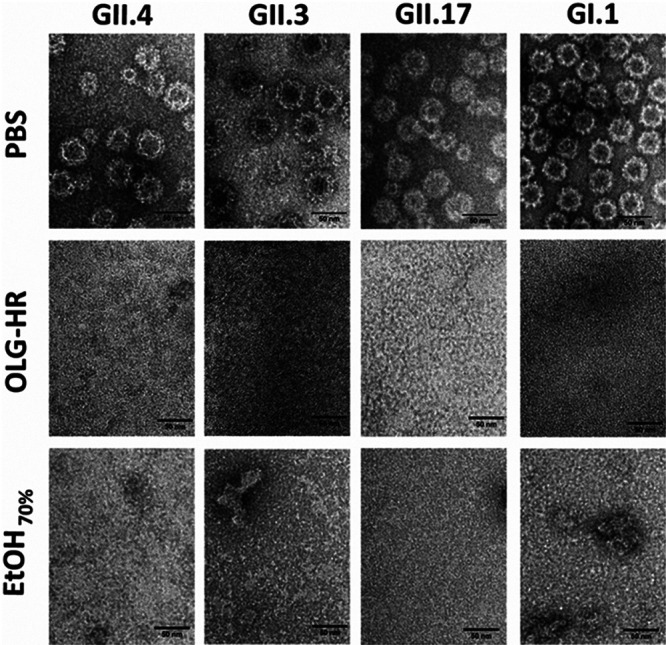
Negative staining electron microscopy. Each HuNoV VLP sample was mixed at a 1:9 ratio of VLP with the indicated treatment for 1 min at room temperature. A 3-μL aliquot of each sample mixture was applied onto a glow discharged 200-mesh 2/2 Quantifoil holey carbon grid containing an 8-nm layer of carbon and incubated for 3 min. Grids were then blotted, washed with Milli-Q water and then 2% uranyl acetate was applied to the grids for 1 min. Finished grids were stored in a dehumidifier. Images were collected at 200 kV on a JEM-2100 electron microscope with a LaB6 filament and a 3k × 4k direct electron detector camera (DE12). Images were collected manually by SerialEM 9 at a ×25,000 magnification. Scale bar, 50 nm.

When samples were imaged using negative stain electron microscopy, there were similar trends as observed in DLS. VLPs treated with PBS alone exhibited icosahedral particles that were approximately 40 nm in diameter (Fig. 6, top row). In contrast, when GII.4, GII.3, GII.17, and GI.1 VLPs were treated with OLG-HR, the electron microscopy (EM) images show only small clumps but no intact VLPs suggesting that OLG-HR induces particle disassembly (Fig. 6, middle row). Based on increased diameters observed in the DLS, it is likely that disassembled products further aggregate into larger-sized clumps. The EtOH_70%_ treatment also results in particle disassembly showing both smaller and larger sized aggregates (Fig. 6, bottom row).

## DISCUSSION

HuNoV is highly contagious and spreads rapidly by fecal-oral, food- or waterborne, and person-to-person transmission (21–23). In addition to causing morbidity and mortality in young children, immunocompromised patients, and the elderly, HuNoV disease causes a substantial economic burden as a result of health care costs and loss of productivity. Unfortunately, effective disinfection methods, except for chlorine (12), to control HuNoV spread are not yet available. Although surrogate viruses were used to evaluate candidate treatments for many years (24–26), the development of a cell culture system to propagate infectious HuNoV in nontransformed human intestinal enteroid monolayer cultures is now allowing therapies and disinfection methods to be directly tested (12, 27). In the present study, we used the HIE cultivation system to evaluate the performance of two virucidal agents, OLG-HR and EtOH_70%_, against HuNoV GII and GI strains.

OLG-HR is a new formula of a disinfectant hand rub that contains olanexidine gluconate and EtOH_70%_. Olanexidine gluconate, developed and marketed by Otsuka Pharmaceutical Factory, Inc. as Olanedine antiseptic solution, was approved by Pharmaceutical and Medical Devices Agency of Japan in 2015 and is used as 1.5% topical solution to prevent postoperative bacterial infections. Olanexidine gluconate is reported to have bactericidal activity against a wide range of Gram-positive bacteria, including MRSA and vancomycin-resistant enterococci, better than that of two other health care antiseptic solutions, chlorhexidine and povidone-iodine (5–7). Olanexidine gluconate-containing hand rub exhibited virucidal activity against murine norovirus and GI, GII, and GIV HuNoV strains when evaluated with culture-independent methods based on reverse transcription-quantitative PCR (RT-qPCR) and RNase or photoreactive intercalators to detect genome derived from intact viruses (16). Recently, OLG-HR has been shown to increase the aggregation of VLPs in a dose-dependent manner (28). However, its activity on human viruses in culture-based methods remained to be determined. Here, we tested the virucidal activity of OLG-HR against HuNoV in a nontransformed HIE monolayer culture system. The virucidal efficacy tests, measured by RT-qPCR, showed that 30 s or 1 min exposure of each of the HuNoV strains to OLG-HR resulted in a complete loss of the ability of virus to bind to the cells, and therefore no viral replication was detected at 24 hpi. In culture-independent assays, Imai et al. (2020) reported that OLG-HR has virucidal activity against GI and GII HuNoV strains based on a reduction in genome copies that was more effective than four other disinfectants tested (ethanol for disinfection, acidity adjusted ethanol (pH 3) for disinfection, base ingredient excluding olanexidine gluconate from OLG-HR, and OLG) (16). The HIE culture system extends these results by allowing functional assessment of viral particle binding to cells and replication. Olanexidine is also reported to interact with bacterial surface molecules, disrupting the membrane integrity and denaturing proteins at relatively high concentrations (5). Our VLP-glycan binding results also indicate that OLG-HR interrupted the binding of VLPs to HBGAs. To understand the mechanism of virucidal activity of OLG-HR, we performed DLS and EM studies on GI and GII HuNoV VLPs. These experiments indicate that the OLG-HR affects the virus by causing particle disassembly and aggregation. It is likely that OLG-HR treatment also causes partial denaturation or induces significant conformational changes in the capsid protein VP1, thereby interfering with the glycan binding. This is in concordance with infectivity results showing that the three GII.4 variants and GII.17 lost the ability to bind to the cells at 1 hpi following OLG-HR treatment.

EtOH_70%_, a second virucidal agent tested in this study, is commonly used as a disinfectant worldwide in the health care field and is effective against most bacteria, fungi, and some viruses (29, 30). The virucidal activity of ethanol on HuNoV remains controversial (31). Previous studies used cultivable surrogate viruses (e.g., murine norovirus [MNV], Tulane virus [TV], and feline calicivirus [FCV]) as alternatives to test the effect of virucidal agents on viral replication, including ethanol (32–34). Using RT-qPCR assays, Tung et al. (2013) reported that the cultivable surrogates (MNV and FCV) do not always mimic HuNoV strains, and common active disinfectant ingredients are relatively ineffective against HuNoV (11). In addition, alcohol susceptibility patterns between different norovirus genotypes are reported to be substantially different, and overall average ethanol sensitivity was similar between GI and GII strains (31). Similar findings were reported by Imai et al. (2020) when they exposed GI and GII strains to EtOH_70%_ (16). In this study, the HIE cultivation system was adopted to assess the effect of EtOH_70%_ on HuNoV infectivity. We found that the effect of EtOH_70%_ on viral infectivity varies among the HuNoV strains. Compared to PBS treatment, EtOH_70%_ at two exposure times (30 s and 1 min) resulted in ≥99% reduction of infectivity of GII.4 Sydney[P31], GII.4_New Orleans[P4], and GII.3, while the infectivity of GII.4 Den Haag[P4], GII.17, and GI.1 after 30 s and 1 min EtOH_70%_ exposure resulted in reductions (86% and 90%, 83% and 87%, and 31% and 81%) of viral infectivity, respectively. Using the same HuNoV cultivation system, Escudero-Abarca et al. (2020) reported the virucidal efficacy of an alcohol-based hand sanitizer against one GII.4 Sydney strain, where moderate reduction in infectivity was observed after 1 min of treatment with 60% EtOH (35). Costantini et al. (2018) also showed that the exposure of GII.4 variants to 70% EtOH for 1 min or 5 min resulted in up to ∼80% reduction of infectivity, but complete virus inactivation was not achieved (12). In fact, Costantini et al. neutralized the effect of EtOH after exposure to the virus by adding 10% fetal bovine serum (FBS) in PBS, while the neutralizer used in this study contains 2 surfactants, Triton X-100 and polysorbate, in addition to other components (Tamol, lecithin, and salts). To understand the virucidal effect of EtOH, these different compounds and formulations would need to be tested further to determine whether they individually or in combination act with EtOH_70%_ resulting in virus inactivation. Our biophysical studies indicate that EtOH_70%_ causes particle disassembly and clumping of the disassembled products. Based on the VLP-glycan binding assay, which shows that EtOH_70%_ treatment does not inhibit glycan binding but rather results in a 2-fold increase in binding with GI.1 VLPs and significant changes in binding with the three other VLPs, it is likely that the glycan-binding site in VP1 remains intact even upon such particle disassembly. This is also consistent with the infectivity assays showing that the EtOH_70%_-treated GI.1 virus retains the capability of binding to the cells but lost the ability to infect. Altogether, the data presented suggest a possible mechanism that OLG-HR and EtOH_70%_ compromise particle integrity and also likely the conformation of the protruding domain of the virus capsid to inhibit interactions with initial cellular receptors like HBGA.

In conclusion, OLG-HR exhibited fast-acting virucidal activity against a broad spectrum of HuNoV strains, including GII and GI strains, and was more effective than ethanol. To our knowledge, this is the first and most comprehensive demonstration of highly effective inactivation of HuNoV infectivity by a hand sanitizer formulation. While the efficacy of the OLG-HR formulation in inactivating HuNoV infectivity is promising, as demonstrated through the applicability of the *ex vivo* HIE system, effectiveness criteria that simulate conditions of the actual use of OLG-HR remain to be proven by examining viral infection control effect through clinical trials. Future studies also might further explore the virucidal activity of OLG-HR in *ex vivo* skin models, on the hands of volunteers, or on solid surfaces that could be modes of transmission of HuNoV.

## MATERIALS AND METHODS

### Preparation of HuNoV-positive/negative stool filtrates.

Stool suspensions (10%) were prepared as previously described (20). PBS was added to GII.4 and GII.3 HuNoV-positive or -negative stools, the mixtures were homogenized by vortexing, and they then were sonicated three times for 1 min. The sonicated suspensions were centrifuged at 1,500 × *g* for 10 min at 4°C. The supernatant was transferred to a new tube and centrifuged a second time, and the resulting supernatant was passed serially through 5-μm, 1.2-μm, 0.8-μm, 0.45-μm, and 0.22-μm low protein-binding syringe filters. The filtered samples were aliquoted and frozen at −80°C for future use in inactivation studies (see Table S1 in the supplemental material). Undiluted and 25% suspensions of the GII.17 and GI.1 stool filtrates, respectively, were prepared as previously described (20, 36). Stool filtrates of all strains were used to evaluate the neutralizer efficiency and virucidal activities of OLG-HR and EtOH_70%_.

10.1128/mBio.02848-21.3 TABLE S1 HuNoV strains tested in this study. Download Table S1, DOCX file, 0.01 MB. Copyright © 2022 Ettayebi et al. 2022 Ettayebi et al. https://creativecommons.org/licenses/by/4.0/ This content is distributed under the terms of the Creative Commons Attribution 4.0 International license.

### Human intestinal enteroid cultures.

Two jejunal, J2 and J4, HIE (jHIE) cultures, secretor positive (sec+) and negative (sec−), respectively, and previously established from surgical specimens from bariatric surgery patients (37, 38), were obtained from an HIE bank maintained by the Texas Medical Center Digestive Diseases Center (TMC DDC) Core. A knock-in J4*^Fut2^* HIE line, established by genetic modification of the sec− J4 HIE line with an active *Fut2* coding sequence, also was used, as it supports enhanced replication for several HuNoV strains (39).

The jHIEs were maintained and propagated in 24-well plates as multilobular cultures in Matrigel as previously described (12, 27). Monolayer cultures in 96-well plates were prepared for infection from the multilobular cultures. Each well of a 96-well plate was coated with 33 μg/mL collagen IV diluted in 100 μL ice-cold water that was removed after 2 h incubation at 37°C. Multilobular HIEs were washed with 0.5 mM EDTA in ice-cold PBS (calcium chloride-magnesium chloride free) and dissociated with 0.05% trypsin/0.5 mM EDTA for 4 min at 37°C. Trypsin then was inactivated by adding complete medium without growth factors [CMGF(−)] (27) supplemented with 10% FBS to the cell suspension. Cells were dissociated by pipetting with a P1000 pipet and passing them through a 40-μm cell strainer. The cells were pelleted for 5 min at 400 × *g*, suspended in a complete IntestiCult proliferation medium (20) with growth factors containing the ROCK inhibitor Y-27632 (10 μM; Sigma), and seeded in a 96-well plate at a concentration of 100,000 cells per well. After 1 day of cell growth as a monolayer, the proliferation medium was changed to IntestiCult differentiation medium to stimulate the cultures to differentiate for 5 days, with the medium being changed every other day (20).

### Human norovirus infection of HIE monolayers.

Five-day differentiated HIE cell monolayers were washed once with CMGF(−) medium and inoculated with HuNoV for 1 to 2 h at 37°C. The inoculum was removed, and monolayers were washed twice with CMGF(−) medium to remove the unbound virus. Differentiation medium (100 μL containing 500 μM bile acid glycochenodeoxycholic acid [GCDCA]) was then added, and the cultures were incubated at 37°C for the indicated times.

### Virucidal agents and neutralizer.

Olanexidine gluconate-containing hand rub (OLG-HR) (16) and 70% ethanol solution (EtOH_70%_) were provided by Otsuka Pharmaceutical Factory, Inc. Each solution was evaluated at 90% (vol/vol) concentration. Neutralizer (40), composed of 1.67% (wt/vol) K_2_HPO_4_, 0.06% (wt/vol) KH_2_PO_4_, 1.17% (wt/vol) lecithin (soybean), 10% (wt/vol) polysorbate 80, 0.5% (wt/vol) sodium thiosulfate hydrate, and 1% (wt/vol) Tamol NN8906 (pH 7.8 to 7.9), was used in this study. Neutralizer was immediately added to the mixture of stool filtrate with the test solution (OLG-HR, EtOH_70%_, or PBS control) after a 30-s or 1-min incubation period at room temperature. The final mixture was immediately diluted in CMGF(−) medium containing GCDCA and added to the cells to test for cytotoxicity and virus infectivity.

### Cytotoxicity assay.

The 3-[4,5-dimethylthiazole-2-yl]-2,5-diphenyltetrazolium bromide (MTT) cell proliferation assay kit (Roche) was used to ascertain whether the virucidal agents and neutralizer were cytotoxic to jHIEs. In a 96-well plate, 5 μL of 10% control (HuNoV-negative) stool filtrate was mixed in triplicate wells with 45 μL of each virucidal agent or PBS (negative control) and incubated at room temperature (see experimental design in Fig. 1A). After a 1-min incubation, 30 μL of each mixture of control stool filtrate with the test solution was immediately diluted 10-fold in 270 μL of neutralizer to inactivate the test substances. Next, the final mixture with neutralizer was diluted further 10-fold in CMGF(−) medium containing 500 μM GCDCA, and 100 μL were inoculated onto 5-day differentiated jHIE monolayers, previously washed once with CMGF(−) medium. After 1 h incubation at 37°C, monolayers were washed twice with CMGF(−) medium, and incubation was continued with 100 μL differentiation medium supplemented with 500 μM GCDCA for 24 h at 37°C. jHIE monolayers were also exposed to 100 μL of 1 mM indomethacin in 0.1% DMSO (positive control to induce cytotoxicity) and to the vehicle (0.1% DMSO) diluted in differentiation medium with GCDCA. Monolayers treated with indomethacin and DMSO were treated for 24 h. At the end of the incubation period, 10 μL of the MTT labeling reagent (final concentration, 0.5 mg/mL) was added to each well. The plate was then incubated for an additional 4-h period to allow intracellular reduction of the soluble yellow MTT to insoluble formazan crystals, followed by adding 100 μL solubilization buffer into each well and incubating overnight at 37°C and 5% CO_2_. The spectrometrical absorbance of the soluble formazan was measured at 570 nm using a microplate reader (SpectraMax M5; Molecular Devices). Each experiment was repeated twice and performed using three technical replicates.

### Neutralizer efficiency assay.

The efficiency of the neutralizer to remove the virucidal effect of the disinfectant was evaluated using the HIE culture system. The virucidal activity of the test agent or PBS (as control) was tested after deactivation with the neutralizer solution prior to adding virus to the mixture (see experimental design in Fig. 2A). Specifically, in a 96-well plate, 5 μL of 10% HuNoV-negative stool filtrate were mixed, in triplicate, with 45 μL of each virucidal material or PBS (negative control) and incubated at room temperature for 1 min. Thirty microliters of each mixture were mixed with 270 μL of neutralizer. Then, 30 μL of this deactivated virucidal compound-neutralizer solution was discarded and replaced with 30 μL of 10% HuNoV-positive stool filtrate previously diluted in CMGF(−) medium with 500 uM GCDCA. A 10-fold dilution was prepared by diluting the final mixture in CMGF(−) medium containing 500 μM GCDCA, and 100 μL was inoculated onto 5-day differentiated jHIE monolayers, previously washed once with CMGF(−) medium. After a 1 to 2 h incubation at 37°C, monolayers were washed twice with CMGF(−) medium, incubated with 100 μL differentiation medium, and supplemented with 500 μM GCDCA for 24 h at 37°C. Virus replication was then assessed in duplicate wells using reverse transcription-quantitative PCR (RT-qPCR). Each experiment was repeated twice and performed using three technical replicates.

### Virucidal efficiency assay.

This assay was performed in the same manner as the neutralizer efficiency assay, except the 10% control (HuNoV-negative) stool filtrate was replaced with a 10% HuNoV-positive stool filtrate (GII.4 Sydney[P31], GII.4 Den Haag[P4], GII.4 New Orleans[P4], GII.3[P21], GII.17[P13], or GI.1[P1]) (Table S1). In a 96-well plate, 5 μL of 10% HuNoV-positive stool filtrate were mixed, in triplicate, with 45 μL of each virucidal agent or PBS (negative control) and incubated at room temperature for 30 s or 1 min. After incubation, 30 μL of each virus mixture was mixed with 270 μL of neutralizer. A 10-fold dilution was prepared by diluting the virus mixture with neutralizer in CMGF(−) medium containing 500 μM GCDCA, and 100 μL was inoculated onto 5-day differentiated jHIE monolayers, previously washed once with CMGF(−) medium. After 1 to 2 h incubation at 37°C, the monolayers were washed twice with CMGF(−) medium, incubated with 100 μL differentiation medium, and supplemented with 500 μM GCDCA for 24 h at 37°C (see experimental design Fig. 3A). Virus replication then was assessed using RT-qPCR. Each experiment was repeated twice and performed using three technical replicates.

### Quantitation of HuNoV RNA replication by RT-qPCR.

Total RNA was extracted from each infected well using the KingFisher Flex purification system and MagMAX-96 Viral RNA isolation kit. RNA extracted at 1 hpi was used as a baseline to determine the amount of input virus that remained associated with cells after washing the infected cultures to remove unbound virus. Replication of the virus was determined by quantifying RNA levels from samples extracted at 24 hpi. For RT-qPCR, the primer pair and probe COG2R/QNIF2d/QNIFS (41) were used for GII genotypes and the primer pair and probe NIFG1F/V1LCR/NIFG1P (42) were used for GI.1 using qScript XLT One-Step RT-qPCR ToughMix reagent with ROX reference dye (Quanta Biosciences). Reactions were performed in duplicates on an Applied Biosystems StepOnePlus thermocycler using the following cycling conditions: 50°C (15 min), 95°C (5 min), followed by 40 cycles of 95°C (15 s) and 60°C (35 s). Standard curves based on recombinant GII and GI HuNoV RNA transcripts were used to quantitate viral genome equivalents (GEs) in RNA samples. The limit of detection of the RT-qPCR assay was 20 and 100 when using recombinant GII and GI RNA transcripts, respectively.

### Production of recombinant VLPs.

The recombinant HuNoV VLPs used in this study were produced by the Protein and Monoclonal Antibody Production Core at Baylor College of Medicine. In brief, the VLPs were produced in HI5 insect cells infected with a recombinant baculovirus that expresses VP1 and VP2 proteins and purified as previously described (43–45). The GenBank accession numbers of recombinant VLPs are as follows: GI.1 (Norwalk) (M87661), GII.3 (TCH-04-577) (AB365435), GII.4 (Sydney) (JX459908), and GII.17 (Katrina-17) (DQ438972).

### Dynamic light scattering.

The hydrodynamic diameters of treated or untreated HuNoV VLPs were measured using dynamic light scattering (DLS) on a Zetasizer Nano instrument (Malvern Instruments, UK). VLP samples in PBS (pH 7.0) were treated for 1 min with OLG-HR, 70% ethanol, or PBS control at a ratio of 1:9, and diluted to 200 nM final concentration prior to DLS analysis. Twelve measurement runs in 3 different experiments were performed with standard settings (refractive Index, 1.335; viscosity, 0.9; temperature, 25°C) for each sample. The average results were created with Zetasizer software.

### Negative stain electron microscopy.

GI.1, GII.3, GII.4, and GII.4 VLPs were diluted to a working concentration of 0.42 mg/mL in PBS, pH 7. Each VLP was then mixed at a 1:9 ratio of VLP to PBS, 70% ethanol, or OLG-HR solution, and each mixture was incubated for 1 min at room temperature. A 3-μL aliquot of each sample mixture was applied onto a glow discharged 200-mesh 2/2 Quantifoil holey carbon grid containing an 8-nm layer of carbon and incubated for 3 min. Grids were then blotted to remove excess fluid, washed with Milli-Q H_2_O, and then 2% uranyl acetate was applied to the grids for 1 min. Finished grids were stored in a dehumidifier at room temperature. Images were collected at 200 kV on a JEM-2100 electron microscope with a LaB6 filament and a 3k × 4k direct electron detector camera (DE12). Images were collected manually by SerialEM 9 at a ×25,000 magnification.

### VLP-glycan binding assay.

VLP-pig gastric mucin (PGM) binding assays were performed as previously described (46). In brief, 96-well plates were coated with PGM (Sigma; 3 μg/mL in PBS, pH 7.2) overnight at 4°C; then the coating mixture was decanted. Each VLP was incubated for 1 min at a 1:9 ratio of VLP to test agent or PBS. The mixture was then diluted 10-fold with PBS (pH 7.2) and passed through a gel filtration column (MicroSporin S-400 HR column; GE Healthcare) to eliminate olanexidine (16). One hundred microliters of the filtered mixture was added in triplicate to PGM-coated wells and incubated for 1 h at 37°C. VLP binding to PGM was then detected by guinea pig polyclonal antiserum to GII.4 Sydney 2012 (for GII.4, GII.3, and GII.17 VLPs) and rabbit polyclonal anti-NV antibody (for GI.1 VLPs) followed by anti-guinea pig and anti-rabbit IgG-horseradish peroxidase antibodies. After a final wash, the bound peroxidase enzyme was detected with 3,3′,5,5′-tetramethylbenzidine (TMB), the reaction was stopped with phosphoric acid, and the absorbance of each sample was determined at a 450-nm wavelength as previously described (46).

### Statistical analysis.

Each experiment was performed twice, with three technical replicates of each culture condition and time point in each experiment. Data from combined experiments are presented. All statistical analyses were performed on GraphPad Prism version 6.0 for Windows (GraphPad Software, La Jolla, CA, USA). Samples with RNA levels below the limit of detection of the RT-qPCR assay were assigned a value that was one-half the limit of detection of the assay. Comparison between groups was performed using the one-way analysis of variance (ANOVA) with Dunnett’s multiple comparison method, when appropriate. *P* values of <0.05 were considered statistically significant.
