# Supplementary figures and images for: Antiviral Activity of Olanexidine-Containing Hand Rub against Human Noroviruses
Source: mBio. 2022 Mar 17;13(2):e02848-21. doi: 10.1128/mbio.02848-21 (PMC9040745; doi:10.1128/mbio.02848-21)

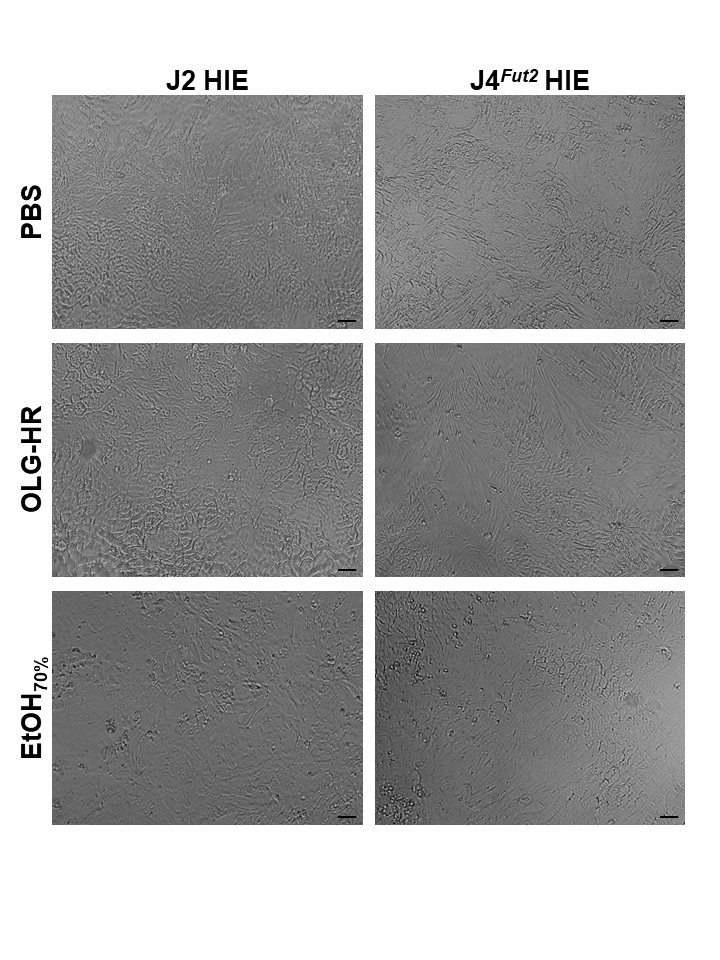

Supplement: FIG S1 [file mbio.02848-21-sf001.tif]

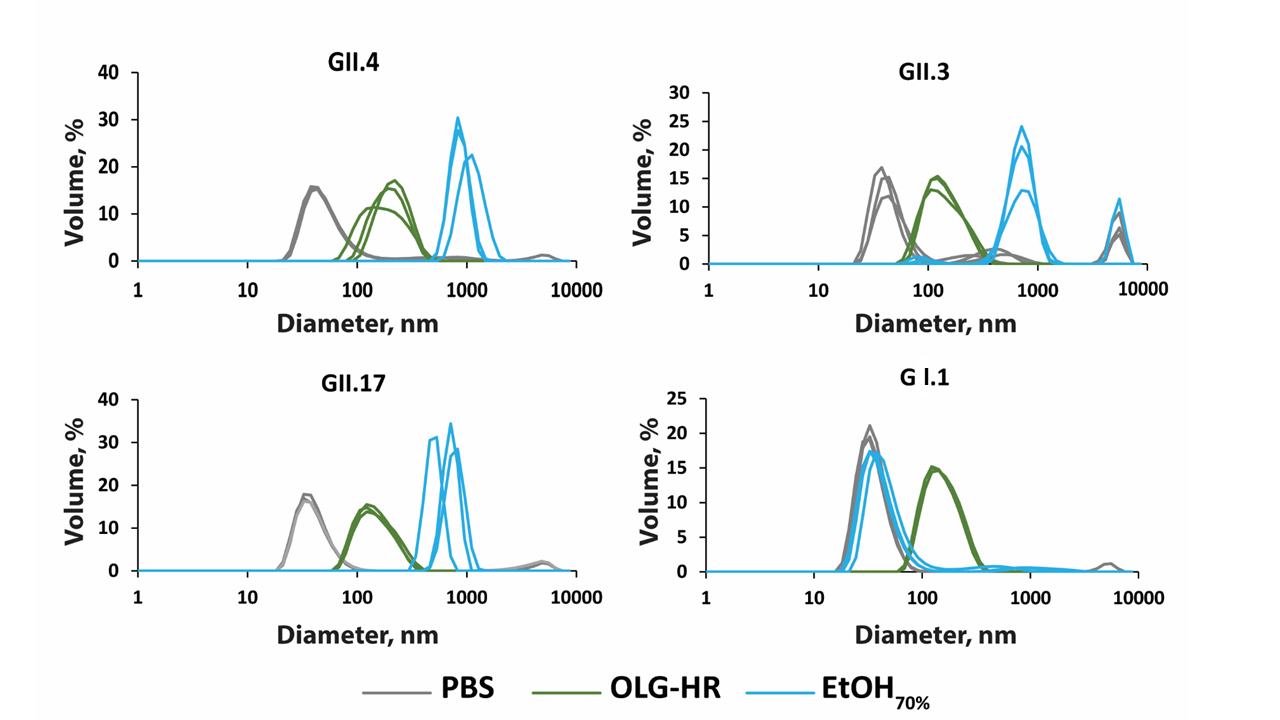

Supplement: FIG S2 [file mbio.02848-21-sf002.tif]
