# Supplementary material for: Antiviral Activity of Olanexidine-Containing Hand Rub against Human Noroviruses
Source: mBio. 2022 Mar 17;13(2):e02848-21. doi: 10.1128/mbio.02848-21 (PMC9040745; doi:10.1128/mbio.02848-21)
Supplement: TABLE S2 [file mbio.02848-21-st002.docx]

| **VLPs** | **DPBS** | **OLG-HR** | **EtOH_70%_** |
| --- | --- | --- | --- |
| **GII.4** | 1.0513 | 0.1056 | 0.8225 |
|  | 1.044 | 0.1178 | 0.771 |
|  | 1.0258 | 0.1172 | 0.7659 |
| **GII.3** | 0.4508 | 0.1242 | 0.5684 |
|  | 0.4174 | 0.1153 | 0.5573 |
|  | 0.4163 | 0.1153 | 0.5606 |
| **GII.17** | 0.5654 | 0.1155 | 0.9024 |
|  | 0.7917 | 0.1264 | 0.8987 |
|  | 0.7584 | 0.1194 | 0.858 |
| **GI.1** | 0.7636 | 0.0646 | 1.5783 |
|  | 0.7144 | 0.065 | 1.5918 |
|  | 0.6955 | 0.0639 | 1.3644 |
