# Supplementary material for: Antiviral Activity of Olanexidine-Containing Hand Rub against Human Noroviruses
Source: mBio. 2022 Mar 17;13(2):e02848-21. doi: 10.1128/mbio.02848-21 (PMC9040745; doi:10.1128/mbio.02848-21)
Supplement: TABLE S1 [file mbio.02848-21-st001.docx]

| Reference Strain | Age (y, m)^#^ | Source of sample | Collection date | Titer (GEs/µl) | TCID_50_ (HIE) |
| --- | --- | --- | --- | --- | --- |
| GI.P1/1968/GI.1/Norwalk | N/A | EC | Apr 2006 | 1.2 x 10^5^ | 2.6x10^3^ (J4*^Fut2^*) |
| GII.P21/GII.3/TCH04-577 | 7 y | Sporadic | Mar 2004 | 8.5 x 10^6^ | 2.0x10^4^ (J2) |
| GII.P4/GII.4 Den Haag/MDA09-01 | N/A | Sporadic | 2009 | 1.1 x 10^7^ | 7.7x10^3^ (J2) |
| GII.P4/GII.4 New Orleans/TCH11-64 | 12 m | Sporadic | Jan 2011 | 3.0 x 10^7^ | 3.6x10^3^ (J2) |
| GII.P31/GII.4 Sydney/TCH12-580 | 17 m | Sporadic | Nov 2012 | 1.8 x 10^7^ | 1.2x10^3^ (J2) |
| GII.P13/GII.17/Katrina-17 | NA | Outbreak | Sep 2005 | 9.3 x 10^6^ | 7.5x10^3^ (J4*^Fut2^*) |
